# Current status of the prognostic molecular markers in medullary thyroid carcinoma

**DOI:** 10.1530/EC-20-0374

**Published:** 2020-10-14

**Authors:** Malgorzata Oczko-Wojciechowska, Agnieszka Czarniecka, Tomasz Gawlik, Barbara Jarzab, Jolanta Krajewska

**Affiliations:** 1 Department of Genetic and Molecular Diagnostics of Cancer, M. Sklodowska-Curie Institute National Research Institute of Oncology Gliwice Branch, Gliwice, Poland; 2 Oncologic and Reconstructive Surgery Clinic, M. Sklodowska-Curie Institute National Research Institute of Oncology Gliwice Branch, Gliwice, Poland; 3 Nuclear Medicine and Endocrine Oncology Department, M. Sklodowska-Curie Institute National Research Institute of Oncology Gliwice Branch, Gliwice, Poland

**Keywords:** medullary thyroid cancer, molecular prognostic factors, RET, RAS, transcriptome

## Abstract

Medullary thyroid cancer (MTC) is a rare thyroid malignancy, which arises from parafollicular C-cells. It occurs in the hereditary or sporadic form. Hereditary type is a consequence of activation of the RET proto-oncogene by germline mutations, whereas about 80% of sporadic MTC tumors harbor somatic, mainly *RET* or rarely *RAS* mutations. According to the current ATA guidelines, a postoperative MTC risk stratification and long-term follow-up are mainly based on histopathological data, including tumor stage, the presence of lymph node and/or distant metastases (TNM classification), and serum concentration of two biomarkers: calcitonin (Ctn) and carcinoembryonic antigen (CEA). The type of *RET* germline mutation also correlates with MTC clinical characteristics. The most common and the best known *RET* mutation in sporadic MTC, localized at codon 918, is related to a more aggressive MTC course and poorer survival. However, even if histopathological or clinical features allow to predict a long-term prognosis, they are not sufficient to select the patients showing aggressive MTC courses requiring immediate treatment or those, who are refractory to different therapeutic methods. Besides the *RET* gene mutations, there are currently no other reliable molecular prognostic markers. This review summarizes the present data of genomic investigation on molecular prognostic factors in medullary thyroid cancer.

## Introduction

**Figure 1:**
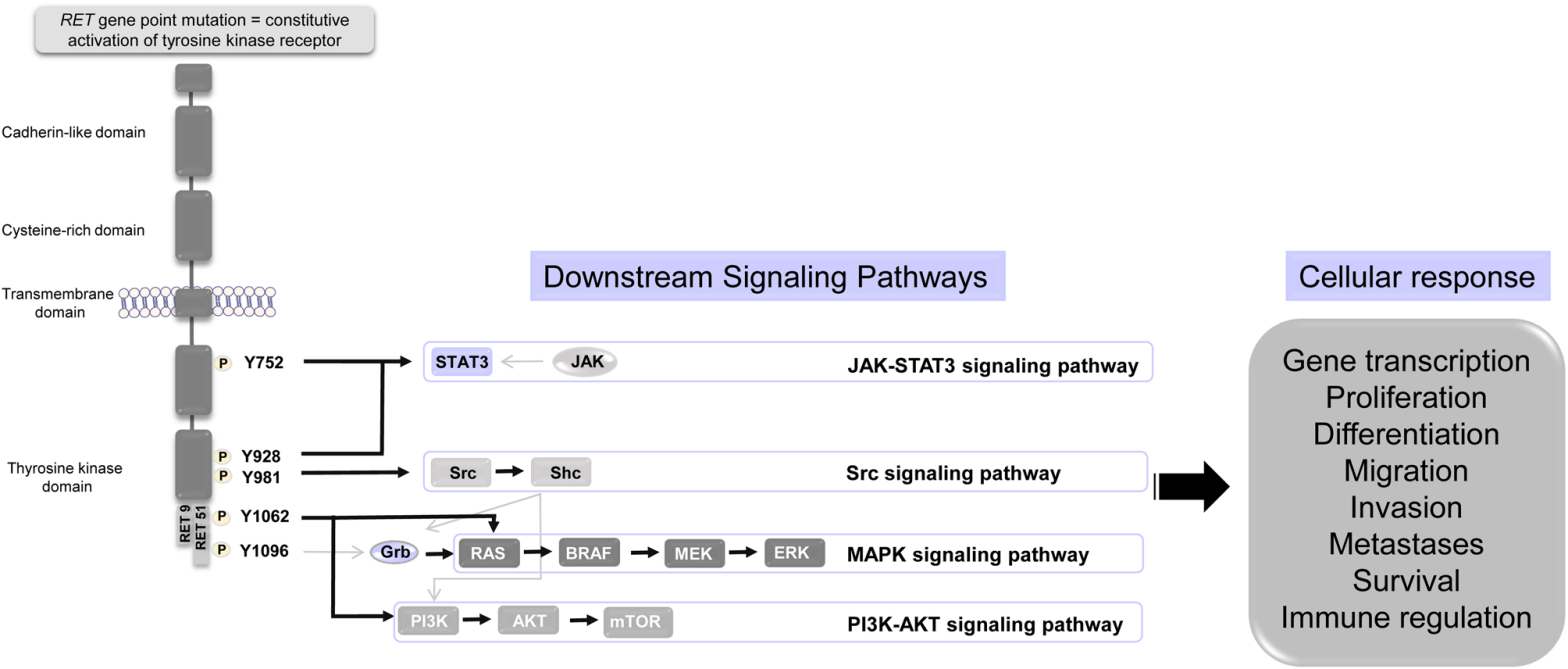
Central signaling pathways activated by *RET* alterations** ** in medullary thyroid cancer. Activation of the RET receptor by different mutations leads to phosphorylation of tyrosine residues (Y) and the recruitment of different downstream signaling pathways. AKT, murine thymoma viral oncogene homolog; BRAF, serine/threonine-protein kinase B-Raf; ERK, extracellular signal-regulated kinase; Grb, growth factor receptor-bound protein; JAK, janus kinase; MEK, mitogen-activated protein kinase; mTOR, mammalian target of rapamycin; PI3K, phosphatidylinositol-3 kinase; RAS, rat sarcoma viral oncogene homolog; RET, rearranged during transfection; STAT3, signal transducer and activator of transcription; Shc, Src (homology 2 domain containing) transforming protein; Src, proto-oncogene c-Src; RET9 a short isoform; RET51 a long isoform.

Medullary thyroid cancer (MTC) is a rare, malignant neoplasm from the C-cell of the thyroid, which occurs in sporadic and hereditary forms. Hereditary MTC develops in the course of MEN2A and MEN2B syndromes due to germline mutations of the RET protooncogene. It accounts for about 20–30% of all MTC cases. Sporadic MTC is mainly related to somatic *RET* mutations, noticed in approximately 50% of cases (1). While 18–80% of *RET*-negative sporadic MTC harbor *HRAS, KRAS*, or rarely *NRAS* somatic mutations (1, 2). The most critical molecular pathways involved in MTC carcinogenesis are shown in Fig. 1.

Currently, following the newest ATA guidelines, a postoperative MTC risk stratification and long-term follow-up are mainly based on histopathological data (primary tumor stage, the presence of lymph node and/or distant metastases), and serum concentration of two biomarkers: calcitonin (Ctn) and carcinoembryonic antigen (CEA) (1). MTC demonstrates a more aggressive clinical course, and it is characterized by a poorer prognosis than differentiated thyroid cancers (DTC). MTC is still diagnosed as a more advanced disease without any significant trend toward earlier stages. Stage III or stage IV is observed in nearly half of the patients at MTC diagnosis (1). Ten-year survival rates for patients initially stratified according to AJCC/UICC TNM classification as stage I, II, III, or IV are 100, 93, 71, and 21%, respectively (1). The most recent study, published by Yeh *et al*., retrospectively evaluated the impact of structural disease progression on overall survival (OS) in patients with metastatic disease at MTC diagnosis. They used tumor volume doubling time (TVDT) as a marker of MTC progression. This analysis involved 43 MTC patients, 33% with lymph node metastases, 23% with distant metastases, and the remaining 44% with both node and distant metastases who were followed for a median follow-up of 11 years (range 2.2–24.0 years). In the whole group, the average TVDT was 1.6 years. The patients were divided into three categories depending on the average TVDT: < 1 year (10 persons), between 1 and 3 years (15 persons), and > 3 years (18 persons). MTC patients with metastatic disease and the average TVDT < 1 year demonstrated a worse prognosis than those with a longer TVDT. The 5-year OS was 100%, regardless of the average TVDT. However, at 10 and 15 years, the OS were 60, 82, 100% and 45, 61, 100% for the average TVDT <1 year, between 1 and 3 years, > 3 years, respectively. The differences were statistically significant (3).

Ctn is a sensitive biomarker, useful both in preoperative diagnostics and postoperative follow-up. Its preoperative level allows the prediction of disease advancement. If serum Ctn is less than 20 pg/mL, there is no risk of lymph node metastases. While Ctn higher than 20, 50, 200, and 500 pg/mL is associated with the ipsilateral central and ipsilateral lateral neck, the contralateral central neck, the contralateral lateral neck, and the upper mediastinum lymph node metastases, respectively (4). The biochemical cure is possible in at least 50% of patients with pretreatment basal Ctn level of 1000 pg/mL or less, but not in patients in whom preoperative Ctn exceeds 10,000 pg/mL (4). The risk of cancer relapse is low, about 4.9% in patients with undetectable postoperative serum Ctn level (5). However, 80% of subjects with palpable MTC and 50% with nonpalpable but macroscopic MTC show elevated serum Ctn level after the operation, despite a radical surgical approach. More than 50% of them demonstrate cancer relapse during a mean 10-year follow-up (6). It has to be emphasized that not only an absolute Ctn value but its dynamics is an even more important prognostic factor. According to published data, shorter Ctn doubling time, less than 6–24 months, negatively impacted recurrence-free and overall survival (1, 3, 7). CEA doubling time has a similar prognostic value (1).

A potential role of serum carbohydrate antigen 19.9 (Ca19.9) as a predictor of mortality in advanced MTC has been demonstrated recently (8). Serum Ca 19.9 level was elevated in 16 out of 100 patients with advanced structural recurrent or persistent disease. Patients showing increased levels of Ca 19.9 had higher serum CEA and Ct concentration and mortality rate compared to the group with normal Ca 19.9 levels (8).

Some papers analyzed a prognostic value of the Ki67 proliferation index in MTC. The Swedish group compared Ki67 in a subset of 12 MTC patients in whom the tissue from primary, metastatic, and recurrent tumors were available. The median Ki67 values of the primary tumor, primary metastasis, and recurrent metastasis were 0.53, 1.11, and 1.65%, respectively. The difference between primary tumors and recurrent metastases was statistically significant. Moreover, the primary tumors in patients with distant metastases, stage III and IV, were characterized by a higher median Ki67 index than primary tumors obtained from patients without distant metastases, stage I, and stage II, 0.51% vs 0.25%, respectively (9). Another study, published by an Italian group, demonstrated that higher Ki67 levels in sporadic MTC were significantly associated with extra thyroid spread, lymph node and distant metastases, advanced stage, and poorer overall survival. Similarly, in the same population, a statistically significant association between *RET* mutations and male gender, tumor size, lymph node and distant metastases, advanced disease, increased risk of persistent disease, and poorer overall survival was observed. So, finally, the authors combined somatic *RET* analysis with Ki67 assessment and showed that patients with somatic *RET* mutation and Ki67 expression level in >50 cells/mm^2^ could identify patients with a high risk of MTC-related death (10).

Even if they allow predicting a long-term prognosis, histopathological or clinical features are not sufficient to select patients showing particularly aggressive MTC courses, requiring immediate treatment or those refractory to different therapeutic methods. The clinical behavior is still unpredictable as some patients may live for years, despite distant metastases. Thus, searching for additional markers is justified. The rapid development of different techniques, observed in the last two decades, allows for analyses of the whole tumor cell transcriptome (microarray-based method), whole exome, or genome (next-generation sequencing; NGS) to identify potential molecular markers. Such stratification, based on molecular markers, has been successfully implemented in daily clinical practice in early breast cancer. MammaPrint test, recommended by the European Society of Medical Oncology and certified by the Food and Drug Administration, based on gene expression profile, allows stratifying the patients as low or high risk of the development of distant metastases. Interestingly, although the *RET M918T* mutation is a known poor prognostic factor, so far, molecular diagnostics in sporadic MTC is not a part of routine clinical practice. The analysis for somatic *RET M918T* or *HRAS, KRAS*, or *NRAS* mutations is even not recommended by ATA as a standard procedure (1). Besides the *RET M918T* mutation, there are currently no other reliable molecular prognostic markers.

This review summarizes the present data of genomic investigation on molecular prognostic factors in medullary thyroid cancer and discusses their potential use in daily clinical practice.

## The*** RET*** gene mutations

Germline mutations of the *RET* gene are linked to differences in the course of hereditary MTC as well as they are essential molecular prognostic factors (1). Hereditary type, diagnosed in 20–30% of patients, is associated with multiple endocrine neoplasia type 2 syndrome (MEN 2), inherited in an autosomal dominant pattern. Depends on the localization of the mutation in the RET proto-oncogene, MEN2 syndrome is currently classified in two distinct variants: MEN 2A and MEN 2B, the last one characterized by a poorer prognosis. However, the vast majority (80%) of MTCs are sporadic. Approximately 50% of them harbor somatic *RET* mutations in tumor cells (2, 11, 12, 13). The most common and the best known *RET* mutation in sporadic MTC, localized at codon 918, is related to a more aggressive MTC course and worse survival (14, 15, 16, 17). The patients harboring somatic RET M918T mutation more frequently had multifocal and larger tumors, lymph node or distant metastases, stage IV disease, persistent disease at the last follow-up than patients with other or without *RET* somatic mutations. A group without somatic RET mutation was an intermediate risk, whereas a group with other *RET* somatic mutations was characterized with the most indolent MTC course (15, 16). Patients without somatic RET mutation demonstrated significantly longer survival than those harboring RET mutation in tumor cells (16). MD Anderson Group demonstrated that circulating *RET* M918T cell-free DNA (cfDNA) could be detected in about 1/3 of sporadic MTC patients with elevated serum Ct level (>100 pg/mL) and advanced disease stages, harboring *RET* M918T mutation in tumor cells. Patients with distant metastases at the time of MTC diagnosis were more likely to have a positive result of the *RET* M918T cfDNA assay. Significant associations with the CEA level, number of metastatic sites, and the treatment outcomes were observed. There was a strong correlation between the detection of *RET* M918T cfDNA and poorer overall survival. Moreover, the detection of *RET* M918T cfDNA allowed predicting a worse outcome better than Ct doubling time (18).

The *RET* mutation M918T predicts a better response for treatment with cabozantinib, a multikinase inhibitor (19, 20). Among all 330 MTC patients enrolled in the phase 3 EXAM trial, 51.2% were *RET* mutation-positive (38.2% with the *RET* M918T mutation), 13.9% were RET mutation-negative, and 4.8% were *RAS* mutation-positive. In 34.8% of patients, RET mutation status was unknown. Cabozantinib showed a beneficial effect on progression-free survival (PFS) compared with placebo in *RET* mutation-positive, *RET* mutation unknown, and *RAS* mutation-positive subgroups. The differences between treatment and placebo arms were statistically significant. On the contrary, in *RET* mutation-negative patients, PFS was similar in treated and non-treated patients. However, the small size of the subgroups and unequal distribution (35 persons – cabozantinib; 11 persons – placebo) may impact the results, so we are not justified to conclude the drug efficacy in these patients. The *RET* M918T positive patients showed the best treatment response among all treated persons. The median PFS value in the cabozantinib arm was 61 weeks, whereas in the placebo arm, only 17 weeks (20). The patients from *RET* M918T positive subgroup are simultaneously the only persons in whom cabozantinib administration significantly prolonged OS; 44.3 months vs 18.9 months in the active and placebo arms, respectively (19). Regarding vandetanib, among patients treated under phase 3 ZETA study, *RET* mutation in tumor cells was present in 52% of patients, no *RET* mutation was not detected in 2.7%, whereas in 45.3%, *RET* mutation status was unknown. A subgroup analysis showed that the treatment in vandetanib was most beneficial in a subgroup of patients with *RET* M918T mutation in tumor cells (21). However, a small number of somatic *RET*-negative patients did not allow to evaluate the efficacy of the drug in these persons.

Mutations of the *RET* gene are usually exclusively present at one specific codon in hereditary and sporadic cases. However, some studies reported double *RET* mutations (22, 23, 24, 25). Double germline *RET* mutations are extremely rare. They were mainly detected as a combination of hot-spot mutation V804M, characteristic for MEN2A, and other affected RET codon mutation not related to MEN2 that may result in an atypical MEN2B phenotype and more aggressive behavior of MTC (1). Only one tandem *RET* mutation with V804M alteration have been reported in familial MTC (FMTC), a variant of MEN2A. Double *RET* mutations detected in other residues than at codon 804 caused an unusual MEN2 phenotype (26, 27, 28). Multiple *RET* mutations were also rarely noticed in sporadic MTC and led to a worse outcome (29). The *RET* V804 mutation, as a second *RET* mutated allele, is responsible for primary resistance to vandetanib (30). Interestingly, it may also cause a secondary resistance to vandetanib, probably related to single tumor cell clones carrying the *RET* V804 substitution upon a multikinase treatment (30).

The presence of *RET* mutations may impact the expression of tyrosine kinase and indirectly be helpful in the treatment-decision making in patients with advanced disease. The Spanish group demonstrated a high expression of VEGFR2 and VEGFR3 in 57% and 43% of the analyzed 103 MTC samples. VEGFR1, PDGFRB, VEGF, KIT, and MET were present in 34–20% of samples, whereas EGFR was overexpressed only in 10% of samples. MTC samples harboring the *RET* C634 mutation presented a higher expression of VEGRF3 and KIT compared with the *RET* M918T-mutated and non-*RET* mutated tumor samples (31). The results of this study may suggest that molecular stratification is a step forward personalizing in MTC treatment. So far, such molecular tumor evaluation is not a part of routine clinical management. It could be the task for future researches.

To sum up, the *RET* M918T mutation in the tumor cells is the best-recognized molecular factor related to a more aggressive MTC course and worse outcomes. It is also a significant predictor of the response to multikinase inhibitors. Although the evaluation of the MTC somatic mutation profile so far is not recommended in daily clinical practice, it is worth considering in patients with advanced disease before qualification for systemic treatment. It seems of particular importance in the coming era of new, very promising selective *RET*-inhibitors LOXO 292 (selpercatinib) and BLU 667 (pralsetinib).

## Polymorphism of the ***RET*** gene

Identification of different mutations in the *RET* gene in hereditary MTC resulted in a better understanding of the correlation between phenotype and genotype, despite the heterogeneity of MTC across the same type of *RET* mutations (32). Regarding sporadic tumors, *RET* somatic mutations are not present in each tumor, whereas the correlation between the type of mutation and clinical features is not so clear. Several authors investigated single nucleotide polymorphisms (SNPs) and their association with susceptibility for the development, progression, or aggressive behavior of MTC. Some studies demonstrated SNPs as genetic modifiers of the RET protooncogene mutations. Elisei *et al*. showed a higher frequency of *RET* G691S polymorphism in patients with sporadic MTC than in healthy controls (27.8% vs 18.9%, *P* = 0.029). These authors reported a strong co-segregation with *RET* S904S polymorphism (33), which was also noticed in other studies (34, 35). However, a large sample study carried out in the European population (36), and other data (37) did not confirm this observation.

The data related to another frequent SNP variant L769L of the *RET* gene were also inconclusive as some papers demonstrated that L769L modulated the age of MTC onset or disease phenotype (38, 39). In contrast, other studies did not (33, 34, 40). Similar divergent data concern S836S SNP variant (33, 34, 40, 41, 42).

An Italian group reported a prognostic value of V109G polymorphism of the *CDKN1B* gene (43, 44). The authors noticed a better MTC prognosis than a wild type allele when the V109G polymorphism was detected. It could suggest the impact of *CDKN1B* V109G variant on the clinical course of sporadic MTC negative for *RET* mutations.

One should emphasize that population studies require a huge number of patients. Due to the rarity of the disease, it is a real challenge in MTC. It also may be a reason for a non-conformance between studies.

To date, none of the proposed SNPs have been introduced into the clinic, and no large-scale studies have been presented that would confirm the importance of SNPs in the development of MTC.

## The RAS gene mutations

The RAS gene mutations, frequently noticed in different malignancies, are also present in thyroid tumors. They are typical for follicular thyroid neoplasms (45, 46). In total, up to 40% of thyroid tumors harbor mutations in the *RAS* subfamily (*HRAS, KRAS*, and the rarest *NRAS*), without any significant differences between follicular thyroid cancer and adenoma. Some data reported *RAS* mutations as a marker of aggressiveness and poorer prognosis in DTC (47). This suggestion was mainly based on a high prevalence of mutated *RAS* isoforms in aggressive DTCs. Mutated *RAS* was detected in up to 60% of cases of anaplastic thyroid carcinoma (48) and 92% of poorly differentiated carcinomas (49). Interestingly, *RAS* mutations have also been detected in sporadic MTC. Early reports showed the absence or very low prevalence of *RAS* mutations in MTCs (50, 51). Nevertheless, recent studies demonstrated that *RAS* mutations were alternative genetic events to *RET* mutation in sporadic MTCs. Their frequency varied from 0 to 43.3% of all sporadic MTC samples (2, 46, 50, 51, 52, 53, 54, 55, 56, 57, 58). Mutation at codon 61 of the *HRAS* gene was the most frequent one.

A few studies are linking clinicopathological features and *RAS/RET* mutation status. A meta-analysis, including 964 MTC cases from 23 studies, revealed that *RAS* mutations did not predict tumor aggressiveness in sporadic MTC. The presence of RAS mutation was not an indicator for more advanced tumor stage, increased risk of nodal and distant metastases, and tumor relapse (59). On the contrary, *RET* mutations highly correlated with disease aggressiveness. The presence of somatic *RET* mutations was associated with lymph node and distant metastasis, tumor recurrence, and patient mortality (59). Some papers demonstrated the correlation of *RAS* mutations and more indolent tumor behavior (51, 57). However, due to a small number of analyzed specimens, the interpretation of these data should be cautious. Valuable data were provided by the recent analysis of the Italian group carried out in 209 sporadic MTC cases. Informative sequencing data were obtained for 181 patients. *RET* somatic alterations were present in 101/181 cases (55.8%), whereas somatic *RAS* mutations were detected in 44/181 MTCs (24.3%). The most common were *HRAS* mutations, the rarest *NRAS* mutations. To evaluate the clinical significance of mutational status, the patients were divided into four groups: *RET* M918T, *RET* other, *RAS* mutations, and not *RET*/not *RAS*. The presence of any *RET* somatic mutation or *RET* M918T alone showed a significant correlation with the advanced disease stage, higher T category, lymph node, and distant metastases. In contrast, *RAS*-mutated cases were characterized by a better outcome, lower MTC stage, and lower T category (17).

To sum up, *RAS* mutations are the second genetic alteration in sporadic MTC. Some data showed a more favorable outcome in patients with somatic* RAS* mutations compared to MTC harboring somatic *RET* mutations. However, other data did not. Further studies are necessary.

## Fusion genes in MTC

The *RET* gene can also be activated by fusion with various partner genes. *RET* gene fusions are typical for papillary thyroid cancer. However, they could be rarely detected in MTC. Only three reports showed the presence of fusion genes in sporadic MTC. *RET* fusion genes and similarly *EML4-ALK* fusion, characteristic for non-small cell lung cancer, were correlated with a very aggressive MTC course (60, 61).

Due to the low number of detected fusion genes, it is currently difficult to judge their importance as a potential marker in MTC.

## Transcriptome analysis

**Figure 2:**
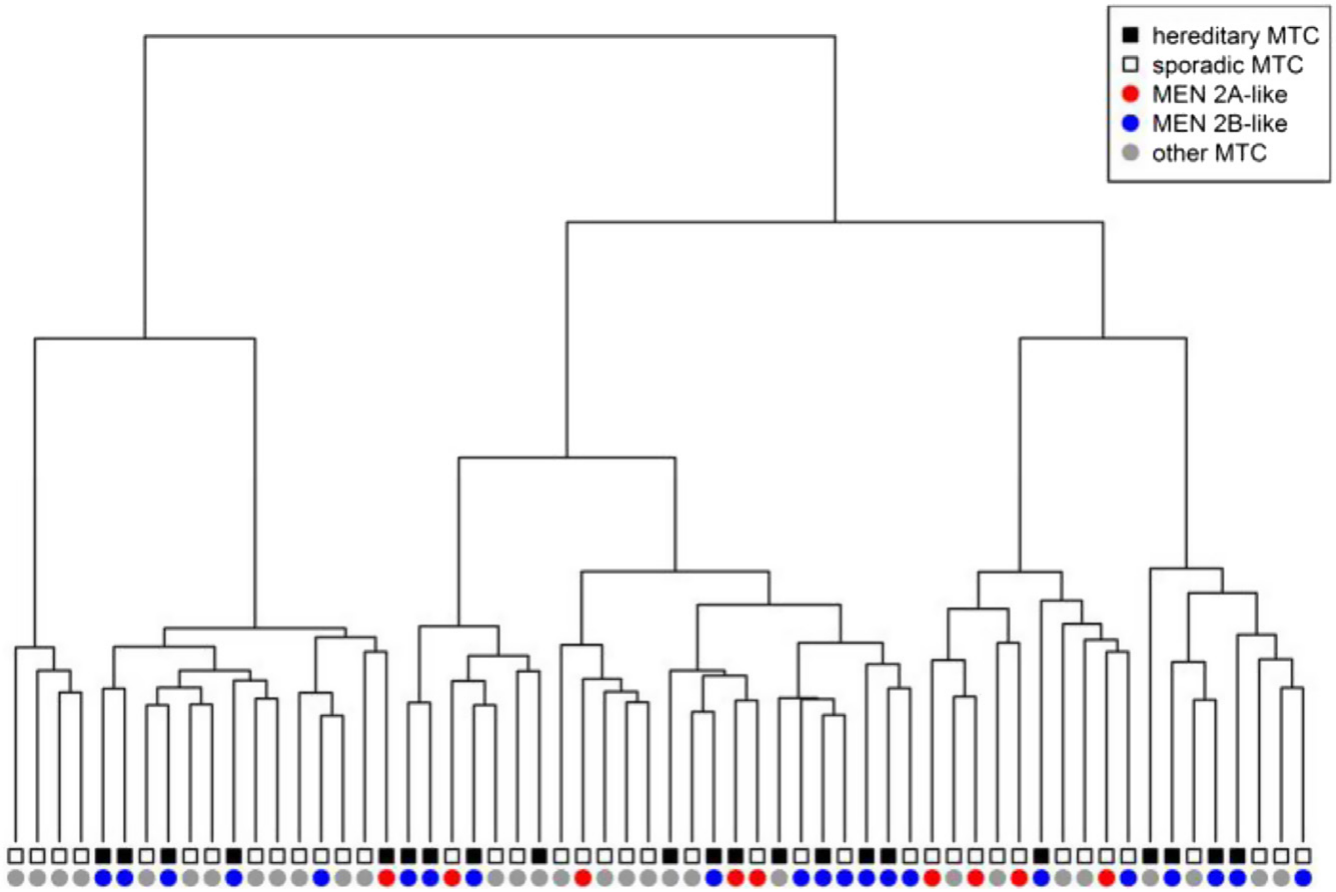
MTC transcriptome heterogeneity. Transcriptomic study showed that differences in the gene expression profile of medullary thyroid cancer are not related to the status and type of RET gene mutations. The source of the observed changes in the transcriptome is still not fully understood and requires further research. MEN2A like – samples with RET mutations characteristic for MEN2A. MEN2B like – samples with mutations characteristic for MEN2B. Reproduced, under the terms of the original CC BY licence, from Oczko-Wojciechowska *et al*., Differences in the transcriptome of medullary thyroid cancer regarding the status and type of *RET* gene mutations, *Scientific Reports* 2017, volume **7**, article 42074 (65).

The analysis of the MTC transcriptome seems to an optimal approach to find MTC molecular prognostic factors. However, one should notice that there is no data regarding the transcriptome of the normal C cell. So, the comparison between normal C cell and MTC is not possible. The studies already carried out compared the transcriptome of more and less aggressive MTC (62, 63, 64, 65). Our analysis of the MTC transcriptome confirmed its very heterogeneous nature (Fig. 2). MTC molecular heterogeneity is clearly reflected in its hereditary form, which presents the variation in the disease phenotype. This phenomenon is associated with different hot-spot *RET* gene mutations. Interestingly RET mutations are also present in sporadic MTC as a somatic mutation in cancer cells. However, there are suggestions that RET mutations are rather related to the progression of the disease but not for its initiation. The other issue is associated with the fact that many *RET*-positive sporadic MTC patients have multiple metastases, but not all metastases present the *RET* mutation (66). Unfortunately, there are no reliable gene expression data showing which genes correlate with a worse prognosis or predict metastasis. The simplest way to resolve this question could be to compare transcriptomes of a sporadic disease with MTC in the course of MEN2A and MEN2B syndrome, the latest one related to a poorer prognosis.

The first study in MTC, aimed to identify the differences in gene expression between sporadic and hereditary tumors, was performed only on 24 samples (67). The authors focused on assessing the source of variability in gene expression profiles and on the evaluation of whether their findings were related to MTC type (sporadic or hereditary). This study revealed some differences in gene expression not related to sporadic/inherited types of disease. These results suggest that gene expression profiles of sporadic and hereditary MTC were very similar (67). Similar data were reported by Ameur *et al*. (62). Analyzing 13 MTC samples, they specified 173 genes differentially expressed between tumors with *RET* mutation at codon 634 and codon 918, however, only two hereditary samples harboring *RET* 634 mutation and 4 with *RET* 918 mutation were included. One of the selected subgroups comprised all samples with germline *RET* 918 mutations and sporadic MTC tumors obtained from patients with lymph node and distant metastases. These data may suggest that the differences in genomic profiling were attributable to MTC aggressiveness. The genes overexpressed in aggressive MTC encoded factors involved in matrix remodeling and cell adhesion, such as *ESM1, COL1A1, COL1A2, CDH11, FAP*, and *CEACAM6*, with the latter one reported as potentially related to cancer cell invasion and metastases (62). The authors also emphasized that the *KAZALD1* gene, playing a role in the proliferation of osteoblasts during bone formation, was also found by other transcriptomic studies as one of the most upregulated genes in MEN2B (63). Jain *et al*. proposed the chondromodulin-1 gene (*CHM1*) as a candidate gene associated with skeletal abnormalities observed in MEN2B patients. High expression of *CHM1* in this paper was detected in all MEN2B tumor samples from patients with skeletal abnormalities and in 2 MEN2B tumor samples from patients without marfanoid habitus. This study included 13 MEN2A and 10 MEN2B samples. Interestingly, 118 genes showed different expression in MEN2A and MEN2B group, and 5 top genes predicted the classification of all MEN2A and MEN2B (Table 1). The same analysis was performed on 11 sporadic MTC samples, among which 4 harbored mutation at *RET* codon 918. These sporadic MTC samples were not classified according to the type of the *RET* gene mutation. They demonstrated a more heterogeneous gene expression profile, being a mixture of genes specified for MEN2A and MEN2B hereditary MTCs (63).

**Table 1:** Main genes deregulated in gene expression analysis of MTC.

Study	Genes	Comparison
Jain * et al.* (63)	*RGS2, CHM1, RGS1, SULF1, MEIS1, PTPRN, ME1, PNMA2, UCHL1, GPX2*	MEN2A vs MEN2B
Oczko-Wojciechowska * et al.* (67)	*GFRA4, MAOB, GABRR1, OGFR, SYT5,*	Sporadic MTC vs hereditary MTC
Ameur * et al.* (62)	*ESM1, POMC, CEACAM6* and *CEACAM7, GHRL, COL1A1* and *COL1A2, FAP, CDH11, RASGEF1A, FBLN1, SPOCK, CIT*	Aggressive inherited vs aggressive sporadic MTC
Ameur * et al.* (62)	*GEM, NR4A1* and *NR4A2, PCDH11y, PTN, KAZALD1, LAMB2*	Hereditary MTC with RET634 and RET918 mutation
Maliszewska * et al.* (64)	*PROM1, GFRA1, LOXL2, GAL, DKK4*	MEN2A vs MEN2B^a^
Sponziello * et al.* (70)	*EZH2, SMYD3*	Aggressive MTC/analysis only selected genes
Tiedje * et al.* (68)	*BRAF, FGFR2, FGFR3, PDGFRA*,*VEGFC*	Metastatic MTC vs primary tumor
Tiedje * et al.* (68)	*FLT1, FLT4, VEGFB*	Advanced MTC: vandetanib therapy: responders vs nonresponders
Musholt * et al.* (69)	*ANXA2,* RAB11A, *SOD1*, transcripts from mitochondrial displacement loop (D-loop), *GNG2, CHGH*	Sporadic MTC vs normal thyroid/analysis only selected genes
Oczko-Wojciechowska * et al.* (65)	*NNAT, CDC14B, NTRK3*	MEN2A vs MEN2B*

^a^MEN2B, germline and somatic mutation in codon 918 of RET gene; MEN2A, germline and somatic in codon 634 of RET gene.

The study of Maliszewska *et al.*, based on the analysis of 41 tumor samples, in contrast to previous ones, showed the differences in gene expression profile between sporadic and hereditary MTCs. Besides, significant differences were observed in the gene expression patterns between tumors with *RET* 634 and *RET* 918 mutations. Four genes were found to be related to MEN2B mutation and one gene with MEN2A (Table 1) (64). However, the results obtained by Maliszewska *et al.* have never been validated by independent studies.

Recently our group published the results of the whole genome analysis, including 60 MTC samples (65). This study confirmed the lack of significant differences between sporadic and hereditary MTC, reported in our preliminary data (67). Only three genes, differentially deregulated between MEN2A and MEN2B samples, were identified. These results supported the opinion that similar pathways might be activated in MEN2 and sporadic MTC (Table 1). One of the selected genes was a tyrosine kinase receptor (*NTRK3*), which showed a high expression in samples with MEN2B mutation and only in one sample with MEN2A mutation. The homogenous gene expression profile might suggest a significant role of epigenetic regulation in MTC development (65).

MTC is a rare thyroid cancer. Probably, therefore, there is still a lack of well-represented studies linking molecular findings with the MTC clinical course to establish prognostic molecular markers. All whole transcriptome studies focused mainly on the biological nature of MTC and possible differences among different *RET* mutations, which could explain the heterogeneity of the MTC phenotype.

Few gene expression studies focused on searching for molecular prognostic markers in MTC. Their most notable limitation is a low number of investigated patients and a selected panel of genes. In a study published by Tiedje *et al*., the gene expression profile of 33 tumor-cell and endothelial-cell tyrosine kinases was analyzed in 32 progressive MTC tumors (68). Four genes showed different expression between samples coming from patients with lymph node and distant metastases and samples from patients who did not develop metastasis (Table 1). Moreover, the expression of 3 genes (*FLT1, FLT4*, and *VEGFB*) was associated with a better response to vandetanib.

Musholt *et al*. analyzed eight sporadic MTC samples (5 with *RET* gene mutations) and their corresponding normal tissue (69). Genes associated with cell proliferation or tumor progression, such as annexin A2, *RAB11A*, trefoil proteins, superoxide dismutase (*SOD1*), mitochondrial displacement loop (D-loop), G protein subunit gamma 1, and chromogranin (*CHGH*) were deregulated in MTC compared to normal thyroid. However, one should remember that MTC originates from C cells, so parafollicular cells should be used as a control. Unfortunately, C cells represent only about 1% of normal thyroid mass, so a comparison between primary tumors and normal C cells is not possible. The question is whether the use of normal thyroid tissue, consisting of mainly follicular cells as control, is justified.

Another study investigated the expression of a gene panel, known as epigenetic regulators by TaqMan low-density arrays, in a series of 54 MTC samples (13 hereditary and 41 sporadic) (70). A significant increase in the expression of histone methyltransferases *EZH2* and *SMYD3* was reported in more aggressive MTC cases (i.e., in patients with lymph node and distant metastases, the persistent disease after primary treatment or disease-related death) and proposed as a prognostic marker for an aggressive disease. The expression of *EZH2* and *SMYD3* genes did not correlate significantly with *RET* or *RAS* mutational status.

The most recent analysis, published in 2019, evaluated the expression of two genes involved in cell transformation and tumorigenesis, which were also overexpressed among others in thyroid cancers: *PTTG1* (pituitary tumor transforming 1) and *AURKA* (aurora kinase A) in 71 sporadic MTC samples. Both genes showed a higher expression in MTC tumor cells comparing to normal thyroid. Moreover, there were significant differences in *PTTG1* expression between MTC stage I or II and stage III or IV and between patients with MTC remission and persistent disease. In contrast, no significant differences were found regarding the presence of distant metastases, serum Ct level, and *RET* mutations. A significant correlation between the expression of both genes was observed (71).

Transcriptomic studies have confirmed the heterogeneous nature of MTC, showing differences in gene expression among samples in both hereditary and sporadic forms. The differences were not related to the type of *RET* mutation or to the genetic background (hereditary vs sporadic). Their nature needs to be explained.

### Next generation sequencing (***NGS***) in MTC

Currently, NGS is routinely used in molecular diagnostics of the whole exons of one gene (e.g., the *RET* gene mutations) or a few selected genes involved in developing the disease. Surprisingly, the first extensive study of a whole coding sequence (exome) in sporadic MTC revealed no other driver mutations than *RET, HRAS*, and *KRAS* (52). Seventeen sporadic MTCs were included in the study and a set of independent 40 MTC samples for validation. Almost 91% of all analyzed samples harbored *RET* and *H/K-RAS* mutations. The *MDC1* gene mutations were found in 1 case by NGS and in 2 specimens in the validation MTC set. Importantly, tumors with *MDC1* mutations were reported as probably more radiosensitive (72).

Kelly *et al*. performed an exome analysis of tumor MTC sample metastasized to bone marrow (73). They found a non-synonymous missense mutation in the *CDKN2C* gene in a primary tumor as well as the loss of heterozygosity, which was identified in *ALK, ATM, CSF1R, GNAS, SMARCB1*, and *NOTCH1* genes. This alteration was not previously reported in metastatic MTC.

Spoziello *et al*. detected germline p.Arg417Gln mutation in the *MET* gene in siblings diagnosed with MTC and other primary cancers (prostate and breast) (70). After a follow-up of 4 and 5 years, no sibling demonstrated evidence of recurrence, distant metastasis, or new primary tumors. The *MET* gene mutation has been previously found neither in hereditary nor in sporadic MTC. However, it is a known cancer driver gene linked to another familial neoplastic syndrome (hereditary papillary renal carcinoma, type 1). No *MET alt*erations were found in the other 13 sporadic MTC without *RET* and *RAS* mutation. Interestingly, cabozantinib, which demonstrated the activity against MTC, also inhibits MET kinase.

Analyzing the NGS data in sporadic MTC, the Italian group evaluated the Variant Allele Frequency (VAF) of the mutated allele within the sample. They reported a correlation of the VAF value with the tumor size and disease outcomes. Larger tumors harbored mutations with a significantly higher VAF value. However, according to the type of mutation, subgroup analysis showed this dependence only in the *RET*-mutated cases but not in *RAS*-mutated ones. Moreover, a higher VAF value of the driver mutation was also associated with a poorer outcome – as it was noticed in patients with metastatic disease compared to disease-free patients (17).

The lack of significant discoveries related to the mechanism of development of sporadic medullary thyroid carcinoma, despite the use of the NGS technique enabling the sequencing of many thousands of genes, full exome or genome, is related to two aspects. It was associated with the rarity of the disease and, consequently, with a too-small number of tested samples. The second one results from a relatively high cost of testing. There might also be a third issue related to the other mechanisms involved in MTC development, not associated with the DNA sequence alteration.

## MicroRNA (miRNA) as potential molecular markers

Recently, microRNA (miRNA), endogenous non-coding small RNAs with lengths ranging from 19 to 25 nucleotides, were identified as essential drivers for tumor development and thyroid cancer progression. They play a significant role in the posttranscriptional regulation of gene expression through mRNA cleavage or translational repression. A considerable advantage of miRNA testing is the possibility of poor quality material such as formalin-fixed paraffin-embedded tissue blocks. A large-scale analysis of miRNA in MTC for the first time was performed in 2008 by the Nikiforova group (74). However, this study was related mainly to follicular-derived thyroid tumors and included only 2 MTC cases. The other studies, based on a larger MTC cohort, specified miRNAs associated with metastatic MTCs and proposed them as molecular prognostic markers. Interestingly, selected miRNA classifiers in these studies are different, which may be either due to the use of distinct methods or patient selection criteria. Romeo *et al*. proposed miR-375 as a strong prognostic factor of poor prognosis in MTC (75), whereas the Santarpia group demonstrated a potential role of miR-200 as a marker of metastatic and drug-resistant MTCs (76). Another study showed significantly downregulated expression of miR-224 in patients with high serum Ct level at MTC diagnosis, advanced MTC stage, persistent or progressive disease, or those who died due to MTC. Interestingly, there was a significant positive correlation between miR-224 expression and somatic *RAS* mutations in tumor cells (77). However, the use of larger cohorts for the validation of these results is necessary.

The application of miRNA analysis as a predictive factor requires validation on a much larger clinical material. Due to the short sequence of miRNA, it can be analyzed in archival material (paraffin block), which is easily available and does not require special preparations or conditions, as testing of fresh tissue. However, currently, such tests are not applicable in daily clinics in MTC.

## Proteomic studies in MTC

Proteomic techniques offer an unbiased platform for the comprehensive analysis of the whole proteome. To date, there are only a few reports devoted to an extensive proteomic scale analysis in MTC. Similarly to the whole transcriptome analysis, proteomic studies are focused on the biology of MTC (78, 79, 80, 81). The recently published proteomic analysis involved three sporadic MTC tumors and corresponding normal tissue (82). The authors selected six among 338 differentially expressed proteins for validation by Western Blot and 2 for immunohistochemistry validation. The validation was done on an independent set of 47 paraffin-embedded archival sporadic MTC specimens. All selected genes (*THBS1, MMP9, RPS6KA3, FN1, SYT1*, and* CEACAM5*) were confirmed. The *FN1* gene also correlated with clinicopathological characteristics and showed an inverse relationship with tumor and lymph node classification. Moreover, a multivariate analysis showed that low *FN1* expression in tumor specimens was an independent predictor of poor prognosis. Interestingly, Oczko-Wojciechowska *et al*. also found significant differences in the expression of the* FN1* gene in their preliminary study comparing MTC transcriptome with the gene expression pattern of normal thyroid samples (67). Nevertheless, it should be emphasized that the expression of all proteins in sporadic MTC was compared to normal thyroid specimens. Thus, it is not possible to exclude that detected changes are related to differences between cancer C cells and normal follicular cells.

Large-scale protein characterization requires specialized and expensive equipment, such as a mass spectrometer, not routinely used in diagnostics. In addition, the proteomic test requires a specially prepared sample, which makes it challenging to perform as a routine diagnostic test. Thus, this type of research is still focused only on scientific aspects.

## Conclusions and future directions

To date, the *RET* gene mutations are the only recognized MTC molecular marker. The possibility of assessing mutation in the *RET* gene is one of the greatest achievements in personalized medicine. Prediction of the disease course based on the type of *RET* mutation substantially improved patient care. However, it is limited only to the hereditary MTC type. We are aware that somatic *RET* mutations may influence the response to tyrosine kinase inhibitors. So, it should be reasonable to introduce *RET* mutation analysis in sporadic MTC into routine clinical practice.

Other reliable molecular markers, possibly linked to MTC prognosis, are currently unknown. Although new high-throughput methods have been applied, there is still a gap in our knowledge of molecular markers in sporadic and hereditary MTC.

At the moment, joining the forces of many research groups to test more samples is an important aspect of molecular research on rare diseases. Most of the available studies are based on analyzes of several dozen samples, which in many cases, is insufficient to obtain adequate results and conclusions. Researching in the framework of international consortia will also reduce the cost, which would enable the analysis of a larger number of samples.

## Declaration of interest

The authors declare that there is no conflict of interest that could be perceived as prejudicing the impartiality of this review.

## Funding

This work was supported by the National Centre for Research and Development project under the program ‘Prevention practices and treatment of civilization diseases’ NCBiR (MILESTONE): STRATEGMED2/267398 /4/NCBR/2015.
